# Establishment and characterisation of a new patient-derived model of myxoid liposarcoma with acquired resistance to trabectedin

**DOI:** 10.1038/s41416-019-0550-2

**Published:** 2019-08-14

**Authors:** Ezia Bello, Silvia Brich, Ilaria Craparotta, Laura Mannarino, Sara Ballabio, Raffaella Gatta, Sergio Marchini, Laura Carrassa, Cristina Matteo, Roberta Sanfilippo, Alessandro Gronchi, Paolo Giovanni Casali, Silvana Pilotti, Maurizio D’Incalci, Roberta Frapolli

**Affiliations:** 1 Laboratory of Cancer Pharmacology, Department of Oncology, Istituto di Ricerche Farmacologiche Mario Negri IRCCS, 20156 Milan, Italy; 2 Laboratory of Molecular Pathology, Department of Pathology, Fondazione IRCCS Istituto Nazionale dei Tumori, 20133 Milan, Italy; 3 Laboratory of Molecular Pharmacology, Department of Oncology, Istituto di Ricerche Farmacologiche Mario Negri IRCCS, 20156 Milan, Italy; 4 Medical Oncology Unit 2, Fondazione IRCCS Istituto Nazionale dei Tumori, 20133 Milan, Italy; 5 Department of Surgery, Fondazione IRCCS Istituto Nazionale dei Tumori, 20133 Milan, Italy

**Keywords:** Sarcoma, Cancer models, Cancer therapeutic resistance

## Abstract

**Background:**

Myxoid liposarcoma is a histological subtype of liposarcoma particularly sensitive to trabectedin. In clinical use this drug does not cause cumulative toxicity, allowing prolonged treatment, generally until disease progression. No other effective therapies are available for trabectedin-resistant patients.

**Methods:**

Through repeated in vivo treatment in athymic nude mice, we have obtained a patient-derived xenograft with acquired resistance to trabectedin.

**Results:**

At basal level, the morphology of the resistant and sensitive models did not differ, in keeping with the finding that the transcriptional profiles of the resistant and sensitive tumours were very similar. After trabectedin treatment adipogenesis was induced in the parental xenograft but not in the resistant one, as assessed by pathological and molecular analysis. A defective transcription-coupled-nucleotide excision repair in the resistant tumour due to mutation of the *UVSSA* gene may be implicated in the mechanism of resistance.

**Conclusions:**

This is the first in vivo model of myxoid liposarcoma with acquired resistance to trabectedin. Although further studies are necessary to characterise the resistance mechanisms, this is a useful tool for studying new therapeutic strategies to overcome trabectedin resistance in patients.

## Background

Myxoid liposarcoma (MLS) is the second most common liposarcoma (35–40%). It arises mainly in the deep soft tissue of the extremities at a median age of incidence of 45 years. Histologically it is characterised by a myxoid stroma with a distinctive plexiform vascular network and low cellularity composed of round/oval non-lipogenic cells, lipoblasts and mature adipocytes. Generally, MLS has an indolent clinical progression, but a subset of patients shows a more aggressive behaviour. This high-grade subtype has high cellularity composed mainly of non-lipogenic round cells and few myxoid stroma. High-grade MLS has an unusual pattern of dissemination: main sites of metastasis are serous membranes (peritoneum, pleura, and pericardium), abdominal cavity and distant soft tissues, and bones.^1^

The pathogenesis of MLS is associated with the t(12;16)(q13;p11) or rarely to the t(12;22)(q13;q12) chromosomal translocations, resulting in the expression of FUS-CHOP or EWS-CHOP transcript, respectively.^2^

MLS is more sensitive to chemo- and radio-therapy than other liposarcoma subtypes.^3^ The marine alkaloid trabectedin is very effective in this histotype, achieving a 90% overall control rate (complete response, partial response and stable disease) with a RECIST objective response rate of 50% and a progression-free survival of 17 months.^4,5^ Radiological imaging showed tissue density changes that precede tumour shrinkage in patients with complete response or partial response.

Histological features of tumours surgically resected after trabectedin showed a major pathological response, with cellular depletion, disappearance of the capillary network, deposition of sclerohyaline material and adipocytic differentiation.^4^ One possible explanation for this peculiar response was related to trabectedin’s ability to inactivate the FUS/CHP chimeric protein, thus allowing reactivation of the adipogenic pathway.^6^

Unlike doxorubicin and ifosfamide, no cumulative toxicity was observed with trabectedin allowing long treatments that can be continued until disease progression. At that point no further efficacious therapies are available.

To unravel the mechanisms of the resistant phenotype and develop new therapeutic strategies to overcome trabectedin resistance, reliable preclinical models are needed. We describe the establishment and characterisation of a new patient-derived xenograft that maintains the main histological and biological features of the human disease and, similarly to the clinical picture, becomes resistant after repeated cycles of trabectedin.

## Methods

### Animals

Six- to eight-week-old female athymic nude mice were obtained from Envigo (RMS, Udine). Animals were housed in individually ventilated cages, with sterilised food and water at libitum and handled under specific pathogen-free conditions in the Institute’s Animal Care Facilities, which meet international standards; they are regularly checked by a certified veterinarian who is responsible for health monitoring, animal welfare supervision, experimental protocols and review of procedures.

All experiments were conducted in conformity with the Italian Governing Law (D.lgs 26/2014; Authorisation no.19/2008-A issued March 6, 2008, by Ministry of Health); Mario Negri Institutional Regulations and Policies providing internal authorisation for persons conducting animal experiments (Quality Management System Certificate—UNI EN ISO 9001:2008—Reg. No. 6121); the NIH Guide for the Care and Use of Laboratory Animals (2011 edition) and EU directives and guidelines (EEC Council Directive 2010/63/UE) and in line with the guidelines for the welfare and use of animals in cancer research.^7^

### Drugs

Trabectedin (Yondelis^®^, ET743) and lurbinectedin were kindly supplied by PharmaMar, S.A. (Colmenar Viejo, Spain). They were dissolved in water and further diluted in saline immediately before use. Doxorubicin (SANDOZ clinical formulation) was diluted with water immediately before use.

### Tumour models

The ML017 patient-derived round-cell myxoid liposarcoma xenograft with type I chimera was maintained through serial transplantation in mice, as previously described.^8^ Briefly, tumours from donor mice were cut into small fragments of about 3 × 3 mm that were engrafted subcutaneously (s.c.) in female athymic nude mice under isoflurane anaesthesia. The histological features of the tumours in mice were verified after each passage and compared to the original human sample in order to maintain the clinical relevance of the model.

### Establishment of a xenograft model with acquired resistance to trabectedin

To obtain a xenograft model with acquired resistance to trabectedin (ML017/ET), mice were engrafted s.c. bilaterally with ML017 tumour fragments, as above. Tumour growth was measured with callipers, and the weights were calculated with the formula: length * (width)^2^/2 considering 1 mm^3^ = 1 mg. Once tumour masses reached about 300–400 mg mice were given the first cycle of trabectedin, 0.15 mg/kg i.v. every seven days for three times (q7dx3). They were checked every 7–10 days until tumour regrowth, and then received a second cycle of trabectedin. This scheme was followed until the local toxicity of trabectedin at the injection site (phlebitis) did not permit further treatment. At that moment mice were euthanised, tumours were removed, re-engrafted in new sets of mice and treated until resistance was observed.

For molecular and pathological analysis, tumour-bearing mice were treated with trabectedin 0.15 mg/kg q7dx3. Fifteen days after the last drug dose they were euthanised by placing the animals in the non-prefilled chamber and introducing 100% carbon dioxide. Tumour samples were collected, frozen in dry ice or formalin-fixed and paraffin-embedded for haematoxylin/eosin staining.

### Histological characterisation

The histologic criteria to define the myxoid liposarcoma histotype and its round-cell subtypes are as described in the WHO classification.^9^

### Western blotting

Proteins extracted from the frozen specimens were homogenised in protein lysis buffer, loaded on SDS-PAGE and immunoblotted as previously described.^10^ An Odissey FC Imaging System (LI-COR) was used for acquisition. Primary anti-CCAAT Enhancer Binding Protein Alpha (C/EBPα)(1:150, cat.#sc-9314) and actin (1:500, cat.#sc-1616) were purchased from Santa Cruz Biotechnology (Heidelberg, Germany). Primary anti-UVSSA (1:500, cat.# A09174) was purchased from Biocompare- BosterBio (Pleasanton, California, USA).

### Chromatin immunoprecipitation

Tumour homogenisation was performed by using a Precellys tissue homogenizer (Bertin Instrument, Montigny-le-Bretonneux, France) then chromatin immunoprecipitation was carried out as previously described.^6^ Briefly, immunoprecipitations were done with 10 µg of the indicated antibodies: CHOP (9C8 ab11419, ABCAM, Cambridge, UK), FUS (A300–302A, Bethyl, Montgomery, TX, USA) and anti-Flag antibody (F3165, Sigma-Aldrich, Milan, Italy). After the immunoprecipitation step, the resulting material was washed with the following buffers: once in low-salt wash buffer (0.1% SDS, 1% Triton X-100, 2 mM EDTA, 20 mM Tris-HCl pH 8, 150 mM NaCl), once in high-salt wash buffer (0.1% SDS, 1% Triton X-100, 2 mM EDTA, 20 mM Tris-HCl pH 8, 500 mM NaCl), once in LiCl wash buffer (0.25 M LiCl, 1% NP-40, 1% sodium deoxycholate, 1 mM EDTA, 10 mM Tris-HCl pH 8). Each wash was followed by 1 min centrifuging at 2000×*g* and supernatant was removed. DNA was eluted by adding 120 μL of elution buffer (1% SDS, 100 mM NaHCO_3_) and vortexing slowly for 15 min at 30 °C. After 1 min centrifuge at 2000×*g*, supernatants were transferred into fresh tubes. After adding 4.8 µL of 5 M NaCl, crosslinks were reversed by incubating samples at 65 °C overnight. The next day RNase A and Proteinase K treatment were done together while shaking at 45 °C. The immunoprecipitated DNA was extracted and the DNAs were suspended in 60 µL of water and analysed by quantitative real-time polymerase chain reaction (RT-PCR). Values are reported as the -fold enrichment over the control antibody (Flag).

The RT-PCR primers used for detection of the proximal promoter sequences are summarised in Supplementary Table 1.

### Pharmacokinetic study

A pharmacokinetic study was done to compare the tumour distribution of trabectedin in ML017/ET and ML017 xenografts. Briefly, tumour-bearing mice were given a single i.v. dose of trabectedin 0.15 mg/kg. Mice were euthanised, and samples collected 1, 6 and 24 h after the drug injection. At least three mice were used for each time point.

Trabectedin was determined in plasma, liver and tumour samples by HPLC-MS as previously reported.^11,12^

### Microarray experiment and data quantification

RNA was extracted using a commercial kit (miRNeasy, QIAGEN, Hilden, Germany). 150 ng of total RNA were reverse-transcribed into Cy3-labelled cRNA using a LowInput QuickAmp labelling kit (Agilent Technologies, Palo Alto, CA, USA), and hybridised onto commercial array platforms as previously described.^13^ At least three biological replicates—i.e. tumour samples from different mice—and, where available, technical replicates—i.e. right and left tumours from the same mouse—were used for each treatment. Raw data from Agilent Feature Extraction (AFE) version 11 were pre-processed, removing features marked as unreliable by the scanning software. Arrays were normalised using the ‘quantile’ method,^14^ and batch correction was applied to normalised data. Normalised data were annotated using the Bioconductor annotation package HsAgilentDesign03994.db.

### Differential expression

Differentially expressed genes (DEGs) were determined using a limma (Linear Models for Microarray and RNA-Seq Data^15^ and setting a logFold-Change cut-off at abs (1), and False Discovery Rate^16^ corrected *p*-value 0.05 or less. Comparisons were set as treated versus untreated control for each treatment.

### Functional enrichment

Functional enrichment analysis was done using gene set enrichment analysis (GSEA^17^) comparing gene expression data from each treatment with matched untreated controls for ML017 and ML017/ET independently, using default parameters. Enrichment was evaluated over specific well-defined biological states or processes, defined as hallmark (50 gene sets)^18^ and over 4436 gene sets from the Biological Process of the Gene Ontology,^19^ both retrieved from the Molecular Signature Database (MSigDB, version 6.1). Results were integrated and plotted using the application EnrichmentMap version 3.0.0 of Cytoscape version 3.6.0 with a *p*-value < 0.01, *q*-value < 0.05 and the overlap parameter set at 0.25.

### DNA sequencing and data analysis

gDNA from the tumour specimens was extracted and purified through an automatic nucleic acid purification system (Qiacube, QIAGEN) with the QIAamp DNA Mini kit (QIAGEN). gDNA from FFPE patient samples was extracted and purified with a Maxwell® RSC DNA FFPE Kit (Promega, Madison, Wisconsin, USA) through an automatic nucleic acid purification system Maxwell® (Promega). Before library preparation, the concentration of gDNA was evaluated using Qubit® dsDNA High Sensitivity Assay Kit (Invitrogen, Carlsbad, California, USA) while the quality was established using 4200 Tapestation (Agilent Technologies). gDNA from FFPE was also examined through a real-time PCR assay with Infinium FFPE QC kit (Illumina, San Diego, California, USA) for next-generation sequencing experiments. 200 ng of DNA were sheared on Bioruptor (Diagenode, Seraing (Ougrée), Belgium) then purified with AMPure XP beads (Beckman Coulter, Brea, California, USA). Following the Sure Select XT protocol (Agilent Technologies), libraries were generated using the Bravo automatic liquid handling station (Agilent Technologies). OneSeq Constitutional Research Panel (Agilent Technologies) was used as capture probes to determine structural variants and mutations of 5791 disease-related genes. After the last AMPure XP beads purification, samples were examined for quality and quantity and the sequencing run was done on a NextSeq 500 sequencer (Illumina San Diego, California, USA) using a 2 × 150 high-output flow cell with 15 samples/run.

Raw sequencing data were demultiplexed with *bcl2fastq* conversion software (Illumina). Quality control was done with FastQC (http://www.bioinformatics.babraham.ac.uk/projects/fastqc). Data were analysed with a publicly available pipeline bcbio-nextgen (https://github.com/bcbio/bcbio-nextgen) on a high-performance computing platform. Somatic calling was done with the combination of MuTect2^20^ and VarDict^21^ variant callers, using the matched normal sample. Somatic variants were filtered at 1% of allelic fraction and 25X coverage for ML017 and ML017/ET. CNVkit^22^ was used to evaluate large copy number gain or loss.

### Digital droplet PCR

Mutation and copy number variation assays of UVSSA were done with droplet digital PCRs (ddPCR QX200TM Droplet DigitalTM PCR System, Bio-Rad, Hercules, California, USA). ddPCRs were done using 2× ddPCR Supermix for Probes (no dUTP, Bio-Rad), the corresponding assays (Bio-Rad) and a variable volume of template in a final volume of 20 μL. The reaction mixture was loaded into DG8 cartridges with added 70 μL of oil and used for droplet generation. Droplets were manually transferred to 96-well PCR plate and thermal-cycled as follows: 95 °C for 10 min, 40 cycles of 94 °C for 30 s and 59 °C for 1 min, 98 °C for 10 min [ramping temperature 2 °C/s]. The ddPCR data were analysed with QuantaSoft Analysis pro software 1.0.596 (Bio-Rad). This software determined the copy number by calculating the ratio of the target molecule concentration A (copies/µL) to the sum of reference molecule concentrations B and C (copies/µL) times the number of reference species copies (N) in the human genome (copy number = (A/(B + C))*N). For the mutation assay, fractional abundance was calculated as follows: F.A. % = (Nmut/(Nmut + Nwt))*100), where Nmut is the number of mutant events and Nwt is the number of WT events per reaction. As negative control DNA extracted from a healthy subject blood was used.

In copy number variation assays, two reference gene probes were used to normalise data (*RPP30* and *EIF2C*, commercially available). For the selected gene, primer pair sequences were designed with an online web tool (https://www.bio-rad.com/digital-assays/#/assays-create/cnd) in the selected genomic regions (Supplementary Table 1).

### Real-time PCR

400 ng of total RNA were reverse-transcribed using the High-Capacity cDNA Reverse Transcription Kit (Applied Biosystems, Foster City, CA, USA). Real-time polymerase chain reaction was run in triplicate for each case using specific primers for *UVSSA* (Supplementary Table 1). All reactions were carried out on the 7900HT Fast Real-Time PCR System (Applied Biosystems, Foster City, California, USA) using QuantiFast SYBR Green PCR Master Mix (QIAGEN). Data were normalised using the geometric mean of two housekeeping genes (*B2M* and *G6PD*, Supplementary Table 1). For analysis, we used the 2−ΔΔCt protocol and results were expressed as fluorescence intensity arbitrary unit.

### Pharmacological studies

The anti-tumour activity of lurbinectedin and doxorubicin was investigated in ML017 and ML017/ET xenografts. When tumour burden reached about 300–500 mg, mice were randomly assigned to the treatment group in a 1:1 ratio. Randomisation was stratified by tumour weight. At least seven mice were enrolled in each treatment group. Doxorubicin was injected i.v., 8 mg/kg, every seven days for two times (q7dx2), lurbinectedin 0.2 mg/kg i.v. q7dx3. The anti-tumour activity was expressed as T/C%, where T and C were respectively the mean tumour weights of treated and control groups. Treatment was considered active when T/C < 40%.

### Statistical analysis

For the evaluation of the antitumor activity at least seven mice were enrolled in each experimental group. A power analysis showed that this sample size has an 80% power to detect an effect size of 1.5, assuming a 5% significance level and a one-sided *t*-test. All the mice enrolled in the pharmacological studies were included in the subsequent analysis of the experimental data. The mean and standard error of tumour volume and body weights were determined for all experimental groups. Statistical analysis was done with GraphPad Prism version 7.02 software (GraphPad software, Inc., La Jolla, CA, USA). ANOVA was used to detect any statistically significant differences between treated and control groups. Student’s *t*-test for unpaired samples was used to compare pharmacokinetic and RT-PCR data.

## Results

**Fig. 1:**
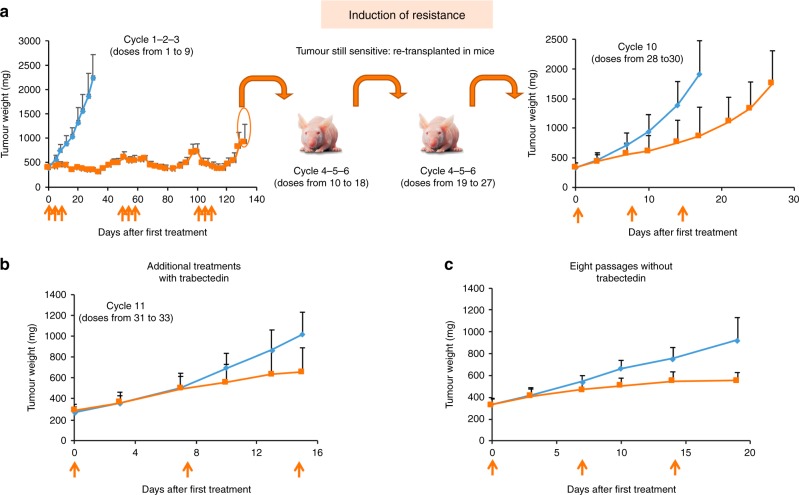
Establishment of the trabectedin-resistant xenograft. ML017-bearing mice were treated with repeated cycles of trabectedin 0.15 mg/kg q7dx3 (orange arrows). Re-transplantation in four different sets of mice and a total of ten cycles of treatment (30 doses) were necessary to obtain a resistant model (**a**). The acquired resistance did not increase with additional treatments (**b**) and was maintained after eight passages without trabectedin (**c**). Blue: vehicles. Orange: trabectedin

To generate a myxoid liposarcoma xenograft with acquired resistance to trabectedin we treated ML017-bearing mice with repeated cycles of trabectedin (Fig.1a). The local toxicity of trabectedin on the tail veins, meant we could not give more than three cycles (nine doses) to the same mouse. Therefore, we needed to transplant tumours relapsing after the ninth drug dose in a new set of mice. Three more cycles (cumulative doses from 10 to 18) were given, but the xenograft was still sensitive to trabectedin, so a further passage in a new group of mice was necessary. During the ninth cycle, no tumour regression or growth arrest was observed. We ran a further passage to verify the sensitivity to trabectedin, and at the 10th treatment cycle (28–30 cumulative doses), tumours were partially resistant. Additional treatments did not appear to increase the acquired resistance (Fig. 1b). The resistant xenograft (ML017/ET) was maintained in vivo for eight passages with no further exposure to trabectedin, and a new efficacy study confirmed the stability of the resistant phenotype (Fig. 1c).

**Fig. 2:**
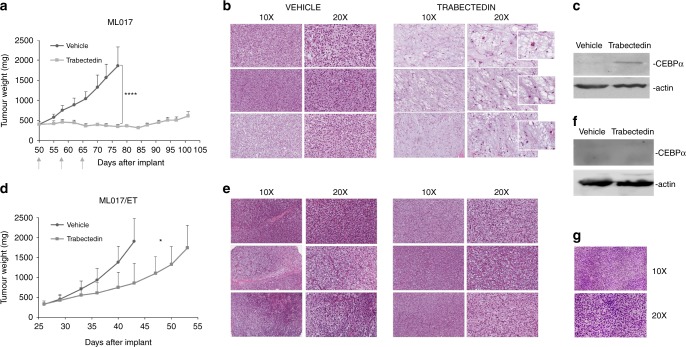
Pharmacological, pathological and molecular responses in ML017 and ML017/ET xenografts. Tumour-bearing mice were treated with trabectedin 0.15 mg/kg q7dx3 (grey arrows). The tumour growth curves showed very good anti-tumour activity in ML017 (**a**) but not in ML017/ET xenografts (**d**). ANOVA followed by Bonferroni’s post hoc test: *p < 0.05 and ****p < 0.0001. In the sensitive model, H&E staining of three different biological replicates showed a decrease of cellularity and of the branching vascular pattern (present although mostly hidden by the cell crowd in the round cell variant); the tumour cells acquired large cytoplasm filled with vacuoles featuring uni-multivacuolated lipoblasts (**b**). The activation of adipogenesis was confirmed by CEBPα expression (**c**). In the resistant model, no relevant changes were observed; hypercellular areas of primitive round cells predominated in both pre- and post-treatment samples (**e**). No CEBPα expression was observed (**f**). **g** Shows representative images of the original human tumour biopsy

Figure 2 compares the ML017 and ML017/ET xenografts. After treatment, ML017-bearing mice showed partial tumour regression followed by long-lasting tumour growth arrest that resulted in a best T/C of 16% on day 80 (panel a). In ML017/ET, however, there was only a small reduction of tumour growth, with a best T/C of 45% on day 43 (panel d). Histologically, no differences were detected between untreated ML017 and ML017/ET xenografts. Moreover, both xenografts were histologically indistinguishable from the original human sample (Fig. 2, panel g). Fifteen days after trabectedin, cellular and vascular depletion coupled with an increase in the stroma mucin pool, and adipocytic maturation were observed in the sensitive model (panel b), while ML017/ET showed no pathological changes compared to control tumours with back-to-back primitive round cells, no intervening stroma and no lipoblast differentiation (panel e). WB detected the expression of cEBPα only in ML017-treated samples, further confirming that trabectedin reactivated adipogenesis in this model but not in the resistant one (panels c and f).

**Fig. 3:**
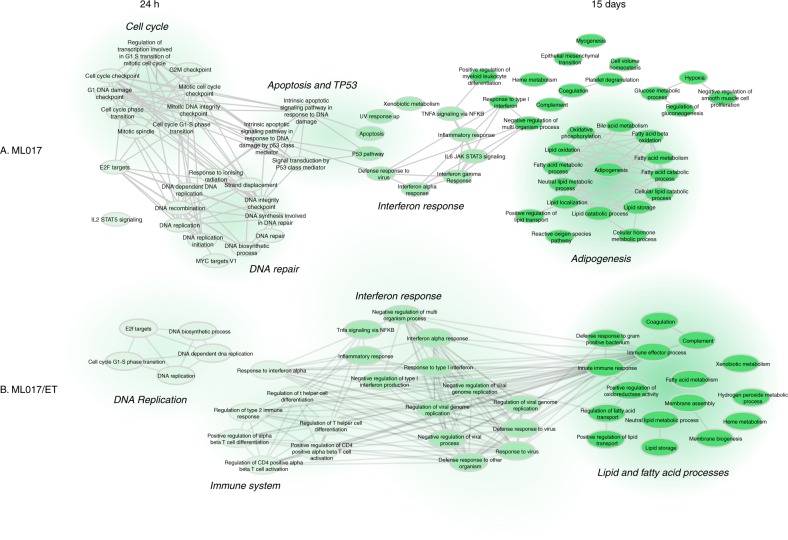
Biological process networks. Functional analysis of ML017 (**a**) and ML017/ET tumours (**b**) 24 h after the first dose and 15 days after the third dose of trabectedin. Functional analysis is described in Materials and methods. Only significant pathways are shown. Light green is related to 24 h, dark green to 15 days. Medium green highlights pathways shared by both 24 h and 15 days. Edges are built based on the presence of genes for each pair of pathways

To further characterise the two models, we did a molecular analysis, investigating the gene expression profiles of the sensitive and resistant models before and after treatment. Supplementary Fig. 1 reports unsupervised cluster analysis and the associated heatmap on ML017 and ML017/ET untreated and treated tumours. Focusing on untreated samples, a differential expression analysis indicated a very low level of differentially expressed genes which could not be associated with any relevant biological process, in keeping with the very similar histological pattern (Fig. 2b, e). After trabectedin the changes in gene expression were different in the sensitive ML017 and the resistant ML017/ET models when compared to matched untreated controls. Figure 3 illustrates the significant pathways related to functional analysis on gene expression of ML017 and ML017/ET 24 h or 15 days after trabectedin. Focusing on ML017, 24 h after the first dose (panel a) we noted an enrichment in functions, such as cell cycle, DNA repair, apoptosis and P53 pathway, suggesting an early cytotoxic effect of the drug with a DNA repair response followed by cell death. By 15 days after the third dose, genes were involved in lipid processes and adipogenesis. The presence of adipogenic pathways together with the expression of the adipogenic marker CEBPα (Fig. 2c) at the same time suggests a late effect of the drug, leading to adipogenic differentiation. On the other hand, with the resistant counterpart ML017/ET at the first time point after trabectedin (Fig. 3b) there was no enrichment in cell cycle, DNA repair or P53 pathways, in line with the low cytotoxic effect. Although 15 days after the third dose, there were changes in some pathways associated with lipid processes, no full adipogenesis was found, unlike in the sensitive counterpart, as demonstrated by the pathological analysis and the lack of CEBPα expression (Fig. 2e, f). Supplementary Table 2 lists the genes differently modulated in ML017 and/or ML017/ET for each shared lipid process.

**Fig. 4:**
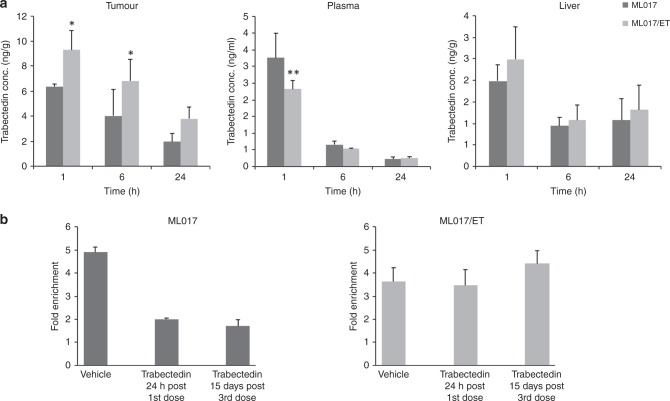
Pharmacokinetic characterisation and ChIP analysis. The pharmacokinetic behaviour of trabectedin was investigated in ML017 and ML017/ET (**a**). Drug levels were slightly higher in the resistant than the sensitive models. Plasma and liver had similar trabectedin concentrations (ANOVA followed by Bonferroni post hoc test, **p < 0.01, * *p* < 0.05). ChIP assay was used to examine the binding of FUS-CHOP on *FN-1* promoter (**b**): the binding of the chimeric protein decreased after trabectedin in the sensitive model but remained stable in the resistant one

Since repeated drug treatment can trigger P-glycoprotein-mediated multi-drug resistance (MDR), we compared the distribution of trabectedin in ML017/ET and ML017 xenografts. The drug concentrations in the resistant tumours were slightly higher than in the sensitive model (*p* < 0.05 at 1 and 6 h) (Fig. 4a). Trabectedin levels in plasma and liver were comparable in the two models. Together with the absence of a differential expression of the *MDR1* gene, this data enables us to exclude the acquisition of the MDR phenotype as mechanism of trabectedin resistance.

As reported by Di Giandomenico et al.,^6^ trabectedin causes detachment of the FUS-CHOP chimera from its target promoters in MLS. We, therefore, investigated the binding of the FUS-CHOP chimera to a well-known target gene, Fibronectin 1, *FN1*, by ChIP experiments in both models. As shown in panel b of Fig. 4, in untreated mice the FUS-CHOP chimera was bound to the *FN1* promoter in sensitive and resistant tumours. In the ML017 xenograft trabectedin caused FUS-CHOP detachment from DNA at 24 h after the first dose, and this was maintained 15 days after the third dose, while in the ML017/ET model the aberrant transcription factor remained bound to the *FN1* promoter at both times. This different behaviour was not due to mutations in the *FUS-CHOP* fusion gene binding site, as demonstrated by Sanger analysis (Supplementary Fig. 2).

**Fig. 5:**
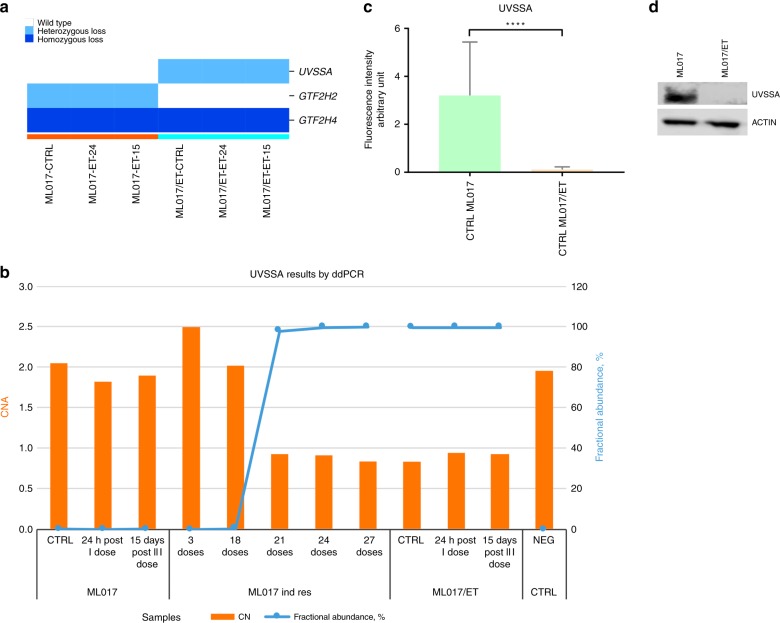
The UVSSA gene in ML017 and ML017/ET xenografts. The genomic sequence of the *UVSSA* gene harboured CNA (heterozygous loss) in the ML017/ET tumours only (**a**). The genomic alterations (CNV and SNV) were orthogonally validated by ddPCR (**b**). Each orange bar indicates the copy number of the *UVSSA* gene (reference y-axis on the right); blue lines indicate the fractional abundance of SNV (reference *y*-axis on the left) calculated as reported in the Material and methods section. The presence of the SNV and the CNA was evaluated in tumour biopsies from ML017-bearing mice after different cycles of exposure to trabectedin (ML017 ind res): the heterozygous loss is detectable after 21 drug doses while the fractional abundance of the SNV increased after 18 drug doses. mRNA and protein levels of the *UVSSA* gene were analysed: in panel **c**, bar graphs show transcriptional expression levels of UVSSA in ML017 and ML017/ET untreated samples. Data show the absence of UVSSA in ML017/ET (orange) untreated samples compared to ML017 (green). These differences were also highlighted by western blot analysis (**d**)

Since trabectedin resistance has been previously associated with nucleotide excision repair (NER) impairment, we analysed genomic defects (i.e., single nucleotide variant, SNV, and copy number alteration, CNA) in genes belonging to the NER pathway by next-generation sequencing (NGS) technology in ML017/ET and ML017 tumours after different exposure times to trabectedin. The genes belonging to NER and NER-related pathways are listed in Supplementary Table 3. The genomic sequence of the *UVSSA* gene, a component of the transcription-coupled nucleotide excision repair (TC-NER), harboured CNA and a SNV in the ML017/ET tumours only. The CNA is a heterozygous deletion seen in all ML017/ET samples, treated and untreated (Fig. 5a). The SNV (c. 256_261del, Supplementary Table 4), has an allelic fraction of 100% due to the presence of only one functional allele in the resistant model. Orthogonal validation by ddPCR confirmed the genomic alterations identified in the *UVSSA* gene (Fig. 5b).

To further evaluate the functional impact of these genomic defects, we analysed mRNA and protein levels of the *UVSSA* gene. Real-time PCR data (Fig. 5c) illustrate the different expression levels of the *UVSSA* gene in sensitive and resistant tumours. These differences were also shown by western blot analysis (Fig. 5d) confirming the impact of the mutation on protein level.

To support the hypothesis that the *UVSSA* gene is involved in trabectedin resistance, we examined by ddPCR the presence of the SNV and the CNA in tumour biopsies from ML017 mice after different cycles of exposure to trabectedin. Figure 5 panel b shows that the heterozygous loss was detectable after the 21st drug dose while the fractional abundance of the SNV increases after the 18th reaching its maximum after the 27th dose. The presence of both events is in line with the acquisition of trabectedin resistance.

**Fig. 6:**
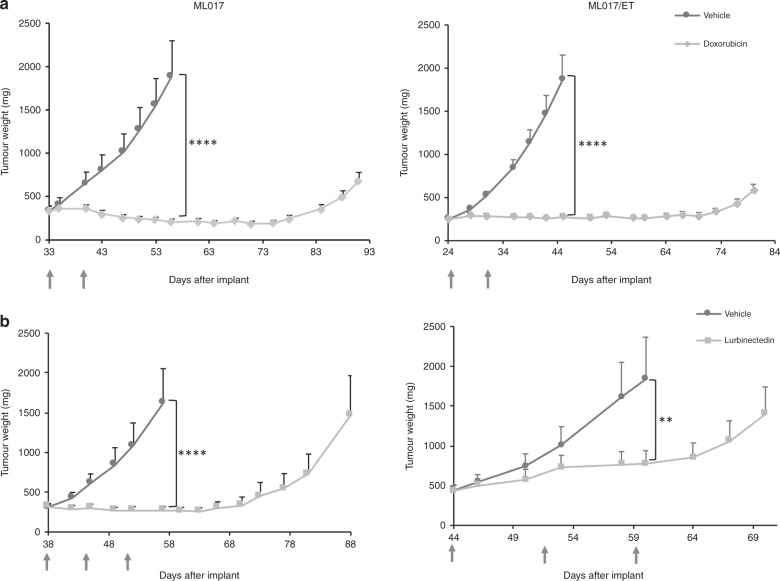
Anti-tumour activity of doxorubicin and lurbinectedin in ML017 and ML017/ET xenografts. Mice bearing ML017 and ML017/ET were treated with doxorubicin 8 mg/kg q7dx2 or lurbinectedin 0,2 mg/kg q7dx3 (grey arrows). Doxorubicin was equally active against the two xenografts (**a**) while lurbinectedin was less effective in ML017/ET than in ML017 (**b**)

To further characterise the pharmacological behaviour of the ML017/ET model, we investigated the sensitivity to doxorubicin (which is clinically used in the first-line treatment of myxoid liposarcoma) and lurbinectedin (a trabectedin analogue with a similar mechanism of action, which is under clinical development). As reported in Fig. 6a, doxorubicin showed very good anti-tumour activity, with growth arrest lasting more than one month after the end of treatment in both models (T/C 11% on day 56 and 15% on day 45). As expected, the efficacy of lurbinectedin was similar to that of trabectedin (panel b), as this compound was very active against ML017 (T/C 17% on day 57) and less effective against the resistant ML017/ET (T/C 42% on day 60).

## Discussion

The present study reports the characterisation of a MLS patient-derived xenograft with acquired resistance to trabectedin. To our knowledge, this is the first MLS model in which resistance to trabectedin was induced through repeated in vivo treatments. The lack of other resistant models is due to the fact that it is very difficult to induce resistance to trabectedin in MLS xenografts. In fact, our original plan was to obtain resistance in several MLS xenografts, but we failed to achieve this as a good response was maintained even after many treatments and we were successful only for one xenograft -ML017/ET­ after ten cycles of trabectedin (corresponding to 30 total doses). The difficulties in inducing resistance to trabectedin in preclinical xenograft models probably reflect what happens in MLS patients as generally their responses to trabectedin last a long time and resistance arises after many cycles.

The resistance to trabectedin of ML017/ET was stable over many passages without further drug treatment but was not complete as trabectedin still induced a partial reduction of tumour volume. However, this was to be expected considering the reported effects of trabectedin on the tumour microenvironment^23,24^ which differently from that in patients, is not exposed to the repeated cycles of drug, being renewed at each passage in mice. The tumour growth rate and the histological features of the resistant ML017/ET were very similar to those of the parental sensitive tumour. However, after trabectedin cellular depletion and adipocytic differentiation were evident in ML017 whereas no histological changes were seen in ML017/ET. This was in line with the anti-tumour activity data, showing regression in the ML017 tumour but not in the ML017/ET subline.

Before treatment, only a few genes were differently expressed in ML017 and ML017/ET, in line with the superimposable morphology. Instead, after trabectedin several changes were found, particularly in genes involved in cell cycle regulation, apoptosis, P53 pathway, DNA repair and adipogenesis. The induction of cEBPα in the sensitive model is in keeping with previous observation in tumour biopsies of MLS patients after trabectedin treatment.^25^ Some of these differences probably underlie the different pathological response. The drug caused diminished cellularity and adipocytic differentiation in the sensitive tumour. In ML017/ET the changes of gene expression seemed less specific and did not lead to phenotypic changes in the tumour tissue.

Different mechanisms of resistance to trabectedin have been described in vitro. The expression of Pgp was observed in cells after prolonged exposure to the drug,^26^ suggesting the possibility that the in vivo resistance might be related to lower drug distribution in the resistant than the sensitive MLS. However, the gene expression and the pharmacokinetic studies with highly specific HPLC-MS excluded this hypothesis, as no difference in expression of the *MDR1* gene was observed and tumour drug levels were comparable to or even slightly higher in the resistant than the sensitive model.

We previously reported that trabectedin displaces the FUS-CHOP oncogenic chimera from some target DNA sequences, suggesting that its activity against MLS was related to this mechanism. We, therefore, speculated that the resistance of ML017/ET might be related either to a mutation of the *FUS-CHOP* binding site sequence or to the drug’s inability to remove the fusion protein from DNA. Sanger sequencing analysis of the DNA encoding for the FUS-CHOP chimera in ML017/ET showed no mutations. Instead, when we examined the binding of FUS-CHOP to a target sequence, *FN1*^25^ by ChIP we found that trabectedin caused the detachment of the chimera only in the sensitive tumour and not in the resistant ML017/ET. A similar finding was already reported for a 402–91 subline resistant to trabectedin after repeated in vitro treatment.^27^ The reason for the different effects in the sensitive and resistant cells is not known but there may be factors that stabilise the complex FUS-CHOP-DNA in the resistant cells. This reasoning derives from a previous observation that in a myxoid liposarcoma naturally resistant to trabectedin, after treatment the chimera tended to reattach more rapidly to DNA consensus sequences than in the sensitive myxoid liposarcomas.^6^

This finding on the *FN1* promoter, chosen on the basis of our previous studies^6,25^ does not translate into a different expression of the *FN1* gene, so the relevance of this mechanism to the resistance to trabectedin must be verified and confirmed on other target genes. ChIP-seq experiments are on-going to address this issue. If successful, they should enable us to establish whether the differences in gene transcription between ML017 and ML017/ET after trabectedin are related to differences in the drug’s ability to modify the binding of FUS-CHOP to target genes.

Previous studies^28,29^ suggested that the loss of functional Xeroderma Pigmentosum, Complementation Group G or Xeroderma Pigmentosum, Complementation Group F was involved in the resistance of some cancer cell lines to trabectedin. These cell lines were hypersensitive to UV light and also to platinum drugs, presumably because NER is involved in the removal of bulky adducts caused by these drugs. The collateral sensitivity to platinum drugs was confirmed when these cell lines were transplanted in vivo.^29^ However, in the ML017/ET tumour, we found no mutations of these two NER genes and the tumour did not change its low sensitivity to platinum drugs (Supplementary Fig. 3). Further analysis of genes belonging to NER and NER-related pathways enabled us to identify a heterozygous deletion and a SNV (c. 256_261del) of *UVSSA*, a component of the Transcription-Coupled NER (TC-NER), with an allelic fraction of 100% only in the ML017/ET tumours.

We can speculate that as TC-NER is particularly important for active genes such as those involved in trabectedin-induced adipogenesis, the loss of function of this repair mechanism may result in a lack of regulation of this pathway. However, we cannot exclude that other mechanisms of resistance are involved. For example, recent data from tumour biopsies from liposarcoma patients related the high levels of the non-histone chromatin protein HMGA1 to tumour aggressiveness and trabectedin resistance.^30^ In vitro HMGA1 depletion restored trabectedin sensitivity in MLS-resistant cells. The higher levels of HMGA1 in ML017/ET than ML017^30^ suggest this mechanism may contribute to causing resistance to trabectedin.

Interestingly, ML017/ET does not show cross-resistance to doxorubicin and this may have clinical implications. A recent finding indicates that trabectedin is as effective as the standard regimen with anthracycline and ifosfamide in MLS in the neoadjuvant setting.^31^ Therefore, if trabectedin is to be used as first-line therapy for this sarcoma—its cumulative toxicity being clearly less than that of the anthracyclines - it is important to know whether relapsing tumours are still potentially sensitive to anthracyclines. Our data suggest that this may be the case.

In conclusion, we describe the first in vivo model of MLS with acquired resistance to trabectedin. Although further studies are needed to completely characterise the mechanisms of resistance, and caution is essential before drawing any definitive conclusion as all data were obtained only in one model of acquired resistance to trabectedin, ML017/ET may offer a useful tool for developing new therapeutic strategies to overcome trabectedin resistance in MLS patients.

## Supplementary information

Supplemental material

## Data Availability

Microarray raw data are available on the ArrayExpress database, under accession ID E-MTAB-2978. Sequence data has been deposited at the European Genome-phenome Archive (EGA, http://www.ebi.ac.uk/ega/), which is hosted by the EBI, under accession number EGAS00001003715.
