# Correction: Establishment and characterisation of a new patient-derived model of myxoid liposarcoma with acquired resistance to trabectedin

**DOI:** 10.1038/s41416-020-0746-5

**Published:** 2020-02-11

**Authors:** Ezia Bello, Silvia Brich, Ilaria Craparotta, Laura Mannarino, Sara Ballabio, Raffaella Gatta, Sergio Marchini, Laura Carrassa, Cristina Matteo, Roberta Sanfilippo, Alessandro Gronchi, Paolo Giovanni Casali, Silvana Pilotti, Maurizio D’Incalci, Roberta Frapolli

**Affiliations:** 1 Laboratory of Cancer Pharmacology, Department of Oncology, Istituto di Ricerche Farmacologiche Mario Negri IRCCS, 20156 Milan, Italy; 2 Laboratory of Molecular Pathology, Department of Pathology, Fondazione IRCCS Istituto Nazionale dei Tumori, 20133 Milan, Italy; 3 Laboratory of Molecular Pharmacology, Department of Oncology, Istituto di Ricerche Farmacologiche Mario Negri IRCCS, 20156 Milan, Italy; 4 Medical Oncology Unit 2, Fondazione IRCCS Istituto Nazionale dei Tumori, 20133 Milan, Italy; 5 Department of Surgery, Fondazione IRCCS Istituto Nazionale dei Tumori, 20133 Milan, Italy

**Keywords:** Sarcoma, Cancer models, Cancer therapeutic resistance

**Correction to:** *British Journal of Cancer* (2019) **121**, 464–473; 10.1038/s41416-019-0550-2; published online 14 August 2019.

Since the publication of this paper, the authors have reported that the Microarray raw data ArrayExpress database accession ID was incorrectly published as E-MTAB-2978. The correct ArrayExpress database accession ID is listed below.

**Data availability**

Microarray raw data are available on the ArrayExpress database, under accession ID E-MTAB-8632. Sequence data have been deposited at the European Genome-phenome Archive (EGA, http://www.ebi.ac.uk/ega/), which is hosted by the EBI, under accession number EGAS00001003715.

