# Supplementary material for: Establishment and characterisation of a new patient-derived model of myxoid liposarcoma with acquired resistance to trabectedin
Source: Br J Cancer. 2019 Aug 14;121(6):464–73. doi: 10.1038/s41416-019-0550-2 (PMC6738121; doi:10.1038/s41416-019-0550-2)
Supplement: Supplementary file 1 — Supplemental material [file 41416_2019_550_MOESM1_ESM.pdf]

Supplementary information:

Figure S1. Heatmap and unsupervised cluster analysis. Normalized gene expression levels of biological replicates of control and treated samples in ML017 and ML017/ET. Rows represent genes. Sample names are reported at the bottom of the heatmap; replicate numbers are indicated in Roman numerals. Unsupervised cluster analysis is shown at the top.

Figure S2. Sanger sequencing on ML017 and ML017/ET.
No mutations were found in either model compared to the human reference. Electropherograms show one of the four binding sites of DDIT3, with no difference between ML017 and ML017/ET. The other three binding sites showed the same concordant sequence (data not shown).

Figure S3. Antitumor activity of cisplatin in ML017 and ML017/ET xenografts.
When tumour weight reached about 300 mg mice were randomized to receive cisplatin 5 mg/kg q7dx3 or saline (arrows). No significant reduction of tumor growth was observed in either the resistant or the sensitive models, suggesting that mutations of NER proteins are not involved in the mechanism of resistance.

Table S1. Primer sequences used for ChIP, RT-PCR and gene sequence regions for ddPCR primers and probe design.

Table S2. Genes involved in the pathways of adipogenesis, fatty acid metabolism, neutral lipid metabolism and lipid storage. ES, enrichment score. Columns headed in orange refer to the ML017 model, in blue to the ML017/ET model.

Table S3. Genes of the TC-NER pathway as reported by the REPAIRtoire Database (Milanowska *et al*, 2011). UVSSA was added manually to the list. Gene annotation with David (Huang *et al*, 2009).

Table S4. SNV in ML017/ET. Somatic SNV with genomic coordinates (hg19 genome build) are described using the HGVS notation.

References

Huang DW, Sherman BT, Lempicki RA (2009) Systematic and integrative analysis of large gene lists using DAVID bioinformatics resources. *Nat Protoc* **4**: 44–57, doi:10.1038/nprot.2008.211.

Milanowska K, Krwawicz J, Papaj G, Kosinski J, Poleszak K, Lesiak J, Osinska E, Rother K, Bujnicki JM (2011) REPAIRtoire--a database of DNA repair pathways. *Nucleic Acids Res* **39**: D788-792, doi:10.1093/nar/gkq1087.

Figure S1

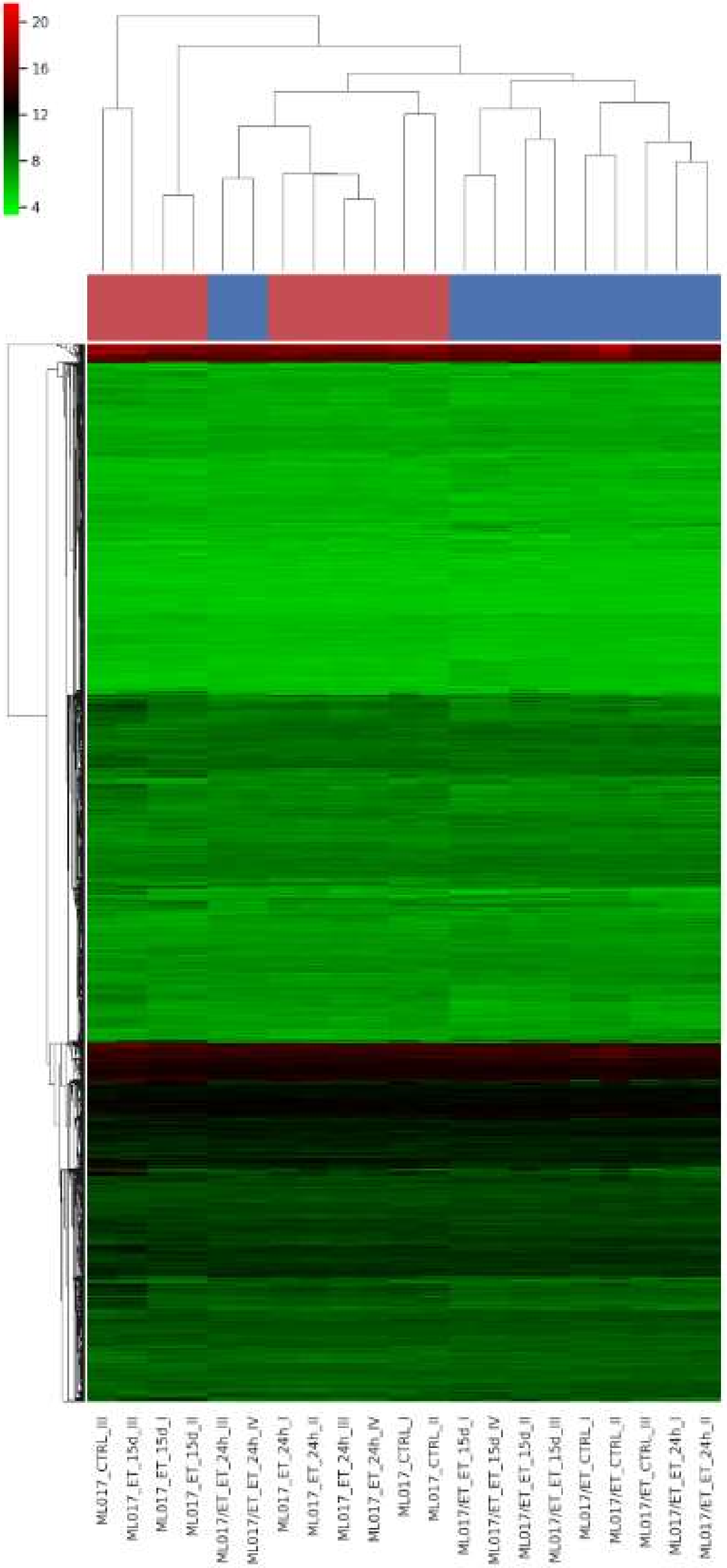
20
16
12
8
4
ML017_CTRL_III
ML017_ET_15d_III
ML017_ET_15d_I
ML017_ET_15d_II
ML017/ET_ET_24h_III
ML017/ET_ET_24h_IV
ML017_ET_24h_I
ML017_ET_24h_II
ML017_ET_24h_III
ML017_ET_24h_IV
ML017_CTRL_I
ML017_CTRL_II
ML017/ET_ET_15d_I
ML017/ET_ET_15d_IV
ML017/ET_ET_15d_II
ML017/ET_ET_15d_III
ML017/ET_CTRL_I
ML017/ET_CTRL_II
ML017/ET_CTRL_III
ML017/ET_ET_24h_I
ML017/ET_ET_24h_II

Figure S2

ML017

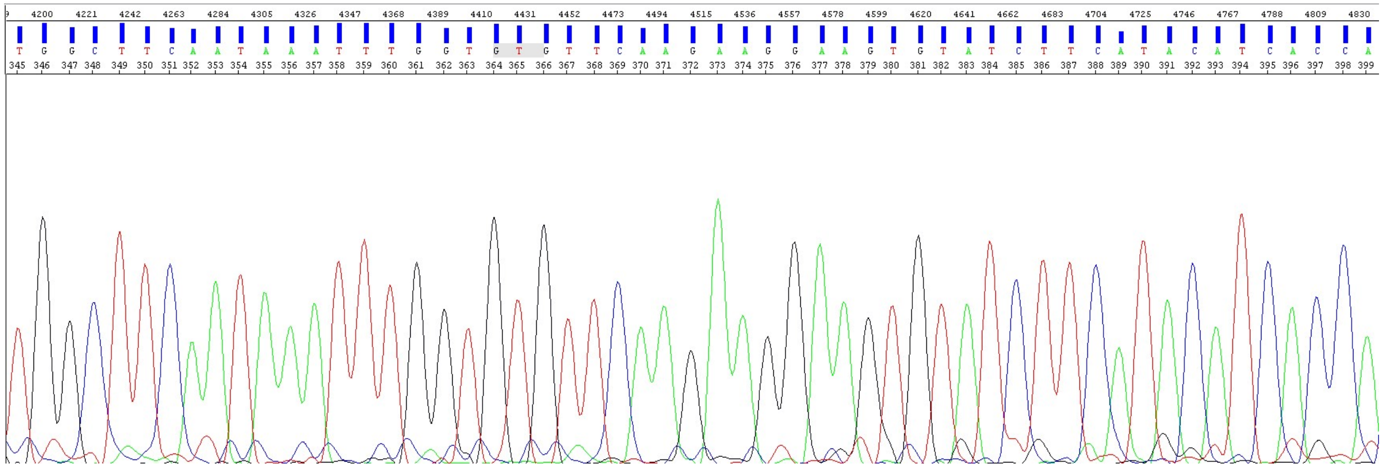

ML017/ET

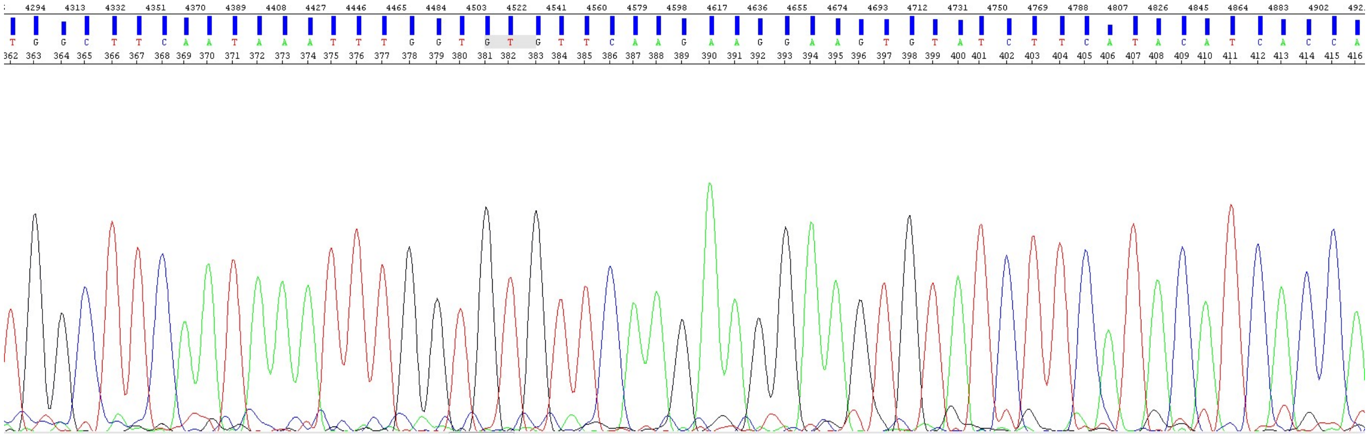

Figure S3

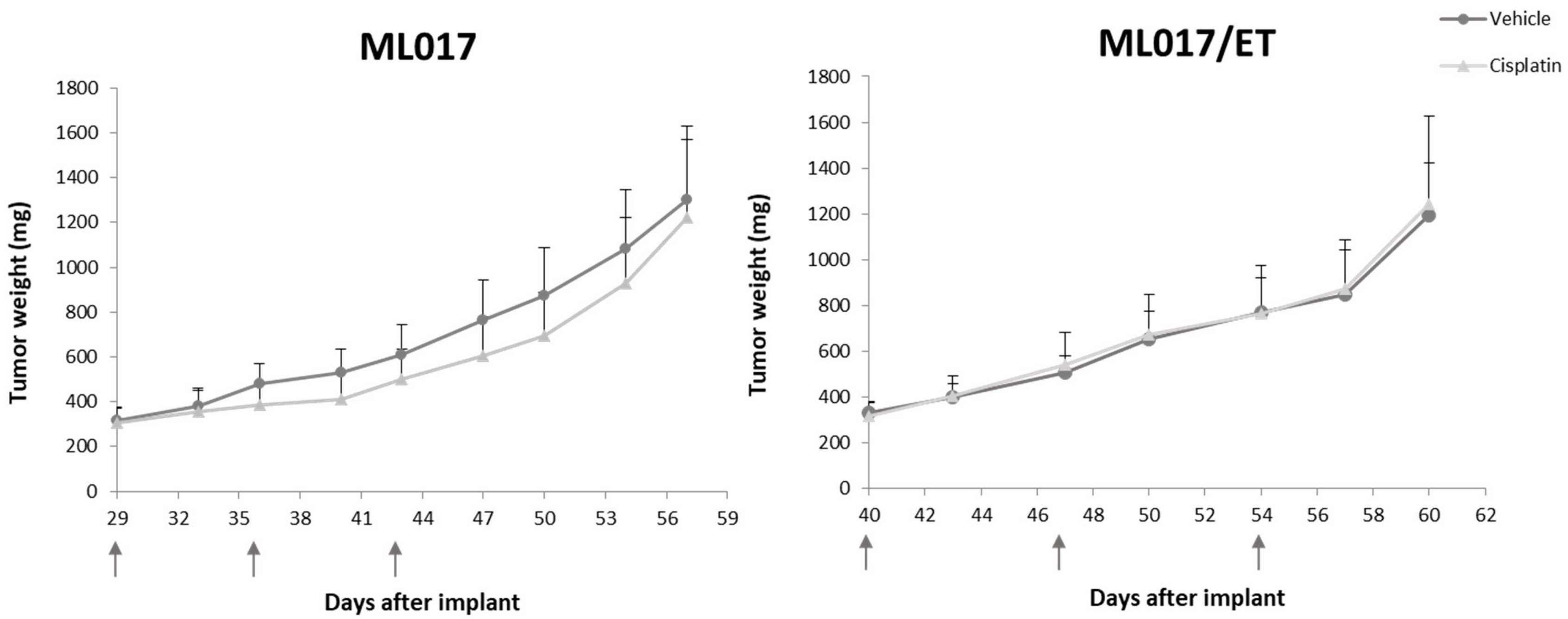
ML017
Tumor weight (mg)
1800
1600
1400
1200
1000
800
600
400
200
0
29
32
35
38
41
44
47
50
53
56
59
Days after implant
ML017/ET
Vehicle
Cisplatin
Tumor weight (mg)
1800
1600
1400
1200
1000
800
600
400
200
0
40
42
44
46
48
50
52
54
56
58
60
62
Days after implant

Supplementary Table 1

Experiment	Gene		EntrezID	Primer Sequence	
Chip	FN-1		2335	FW	CTTCGCTTCACACAAGTCCA
				RV	GCAGCGAACAAAAGAGATGC
	Control satellite		/	FW	CAATTATCCCTTCGGGGAATCGG
				RV	GGCGACCAATAGCCAAAAAAGTGAG
Real-time PCR	UVSSA		57654	FW	CCCTTCTTTCCCCGAGTAG
				RV	ATCCATATCTGGTGGGATGAG
	B2M		567	FW	AAGCAGCATCATGGAGGTTT
				RV	AGCAAGCAAGCAGAATTTGG
	G6PD		2539	FW	TGCCCCCGACCGTCTAC
				RV	ATGCGGTTCCAGCCTATCTG
ddPCR	CNV assay	RPP 30	10556	Region	ATGAGGAACCTGAAACTTCATGTTAAGTAACTTGTAAGTGGTAGTGCATAGACTTTAAATCAGGCAGACTGACACTA GAGTTCACATTCATAACCACTCCTCAAATGTCCTCCTACTCTTGAC
		EIF2 C	26523		GAGGGCTACTACCACCCGCTGGGGGGTGGGCGCGAGGTCTGGTTCGGCTTTCACCAGTCTGTGCGCCCTGCCATGTG GAAGATGATGCTCAACATTGATGGTGAGTGGGGAGAGCTATGGAGC
		UVS SA	57654		TCCGGGCAGGCTGCCTCCGTTAGGGCCGCCCCTGCTCTCCGGACGCGACTTTTCATTGGTCTCAGAATTTCTTGGCTC CTCTTGGCCTCTGCAGCCTTGCTGGAGGCTGCCCTGCGGAATCTG
	Mutational assay	UVS SA	57654		TGGAGGAACTCTTCGTCAGGTCTCACCAGTTCCGGATGCTGGTTGTTTCCAACTTCCAGGA[GTTCCT/-]GGAGCTCACGCTGGGCACAGACCCCGCACAGCCTCTGCCGCCCCCCAGGGAGGCGGCACAG

Supplementary Table 2

Legend	
**Rank metric score**	Score associated to the position of the gene in the ranked list of the gene enrichment analysis.
**Running ES**	The enrichment score at this point in the ranked list of genes as computed by the enrichement analysis.
	ML017
	ML017/ET

Adipogenesis					
**Gene Symbol**	**Description**	**Rank metric score**	**Running ES**	**Rank metric score**	**Running ES**
**ADIPOQ**	adiponectin, C1Q and collagen domain containing	0.75	0.04	/	/
**MRAP**	melanocortin 2 receptor accessory protein	0.56	0.07	/	/
**SLC1A5**	solute carrier family 1 member 5	0.39	0.08	/	/
**RETSAT**	retinol saturase	0.36	0.10	/	/
**CIDEA**	cell death-inducing DFFA-like effector a	0.33	0.12	/	/
**UCP2**	uncoupling protein 2	0.32	0.13	/	/
**LIPE**	lipase E, hormone sensitive type	0.30	0.15	/	/
**LEP**	leptin	0.29	0.16	/	/
**CDKN2C**	cyclin dependent kinase inhibitor 2C	0.28	0.17	/	/
**CD151**	CD151 molecule (Raph blood group)	0.28	0.19	/	/
**PEMT**	phosphatidylethanolamine N-methyltransferase	0.27	0.20	/	/
**C3**	complement C3	0.26	0.21	/	/
**FAH**	fumarylacetoacetate hydrolase	0.25	0.22	/	/
**ABCA1**	ATP binding cassette subfamily A member 1	0.25	0.23	/	/

**SLC25A1**	solute carrier family 25 member 1	0.25	0.25	/	/
**GADD45A**	growth arrest and DNA damage inducible alpha	0.24	0.26	/	/
**ME1**	malic enzyme 1	0.24	0.27	/	/
**GPX3**	glutathione peroxidase 3	0.23	0.28	/	/
**GBE1**	1,4-alpha-glucan branching enzyme 1	0.23	0.29	/	/
**PRDX3**	peroxiredoxin 3	0.23	0.30	/	/
**MGLL**	monoglyceride lipase	0.22	0.31	/	/
**ESYT1**	extended synaptotagmin 1	0.21	0.32	/	/
**REEP6**	receptor accessory protein 6	0.21	0.33	/	/
**DRAM2**	DNA damage regulated autophagy modulator 2	0.19	0.33	/	/
**AIFM1**	apoptosis inducing factor mitochondria associated 1	0.19	0.34	/	/
**MGST3**	microsomal glutathione S-transferase 3	0.19	0.35	/	/
**LTC4S**	leukotriene C4 synthase	0.19	0.36	/	/
**DHRS7B**	dehydrogenase/reductase 7B	0.19	0.37	/	/
**DGAT1**	diacylglycerol O-acyltransferase 1	0.18	0.37	/	/
**PDCD4**	programmed cell death 4	0.18	0.38	/	/
**CYC1**	cytochrome c1	0.18	0.39	/	/
**DECR1**	2,4-dienoyl-CoA reductase 1	0.17	0.39	/	/
**ADCY6**	adenylate cyclase 6	0.17	0.40	/	/
**POR**	cytochrome p450 oxidoreductase	0.16	0.40	/	/
**FABP4**	fatty acid binding protein 4	0.16	0.41	/	/
**SORBS1**	sorbin and SH3 domain containing 1	0.16	0.42	/	/
**SLC19A1**	solute carrier family 19 member 1	0.16	0.42	/	/
**CPT2**	carnitine palmitoyltransferase 2	0.15	0.43	/	/
**SDHB**	succinate dehydrogenase complex iron sulfur subunit B	0.15	0.44	/	/
**PGM1**	phosphoglucomutase 1	0.15	0.44	/	/
**IDH3A**	isocitrate dehydrogenase 3 (NAD(+)) alpha	0.15	0.45	/	/
**AGPAT3**	1-acylglycerol-3-phosphate O-acyltransferase 3	0.15	0.46	/	/
**PHYH**	phytanoyl-CoA 2-hydroxylase	0.15	0.47	/	/
**SNCG**	synuclein gamma	0.14	0.47	/	/
**ARL4A**	ADP ribosylation factor like GTPase 4A	0.14	0.47	/	/

**SUCLG1**	succinate-CoA ligase alpha subunit	0.14	0.47	/	/
**TKT**	transketolase	0.14	0.48	/	/
**ACADS**	acyl-CoA dehydrogenase, C-2 to C-3 short chain	0.13	0.48	/	/
**ACAA2**	acetyl-CoA acyltransferase 2	0.13	0.48	/	/
**ITIH5**	inter-alpha-trypsin inhibitor heavy chain family member 5	0.12	0.48	/	/
**DHRS7**	dehydrogenase/reductase 7	0.12	0.48	/	/
**ACO2**	aconitase 2	0.12	0.49	/	/
**GPX4**	glutathione peroxidase 4	0.12	0.49	/	/
**MYLK**	myosin light chain kinase	0.12	0.50	/	/
**UCK1**	uridine-cytidine kinase 1	0.12	0.50	/	/
**SCP2**	sterol carrier protein 2	0.11	0.50	/	/
**NDUFB7**	NADH:ubiquinone oxidoreductase subunit B7	0.11	0.49	/	/
**LIFR**	LIF receptor alpha	0.11	0.50	/	/
**HADH**	hydroxyacyl-CoA dehydrogenase	0.10	0.50	/	/
**STAT5A**	signal transducer and activator of transcription 5A	0.10	0.50	/	/
**IDH1**	isocitrate dehydrogenase (NADP(+)) 1, cytosolic	0.10	0.50	/	/
**SAMM50**	SAMM50 sorting and assembly machinery component	0.10	0.51	/	/
**APOE**	apolipoprotein E	0.10	0.51	/	/
**CS**	citrate synthase	0.10	0.51	/	/
**ARAF**	A-Raf proto-oncogene, serine/threonine kinase	0.10	0.51	/	/
**UQCR10**	ubiquinol-cytochrome c reductase, complex III subunit X	0.10	0.52	/	/
**IDH3G**	isocitrate dehydrogenase 3 (NAD(+)) gamma	0.09	0.52	/	/
**TOB1**	transducer of ERBB2, 1	0.09	0.53	/	/
**PQLC3**	PQ loop repeat containing 3	0.09	0.52	/	/
**RAB34**	RAB34, member RAS oncogene family	0.09	0.52	/	/
**ADIPOR2**	adiponectin receptor 2	0.08	0.52	/	/
**RREB1**	ras responsive element binding protein 1	0.08	0.52	/	/
**TST**	thiosulfate sulfurtransferase	0.08	0.52	/	/
**ALDOA**	aldolase, fructose-bisphosphate A	0.08	0.52	/	/
**BAZ2A**	bromodomain adjacent to zinc finger domain 2A	0.08	0.53	/	/

**ANGPTL4**	angiopoietin like 4	0.08	0.53	/	/
**PREB**	prolactin regulatory element binding	0.08	0.53	/	/
**APLP2**	amyloid beta precursor like protein 2	0.08	0.53	/	/
**HSPB8**	heat shock protein family B (small) member 8	0.07	0.53	/	/
**ATP5O**	ATP synthase, H+ transporting, mitochondrial F1 complex, O subunit	0.07	0.53	/	/
**SLC27A1**	solute carrier family 27 member 1	0.073522	0.53612494	/	/
**Fatty acid metabolism**					
**Gene**	**Description**	**Rank metric score**	**Running ES**	**Rank metric score**	**Running ES**
**EPHX1**	epoxide hydrolase 1	0.49	0.03	0.18	0.39
**ACSL5**	acyl-CoA synthetase long-chain family member 5	0.37	0.06	0.34	0.26
**RETSAT**	retinol saturase	0.36	0.08	0.21	0.36
**CBR3**	carbonyl reductase 3	0.35	0.11	0.16	0.43
**CA2**	carbonic anhydrase 2	0.35	0.13	0.57	0.13
**CIDEA**	cell death-inducing DFFA-like effector a	0.33	0.18	0.52	0.24
**HPGD**	hydroxyprostaglandin dehydrogenase 15-(NAD)	0.25	0.23	0.32	0.29
**ME1**	malic enzyme 1	0.24	0.26	0.23	0.33
**REEP6**	receptor accessory protein 6	0.21	0.31	0.28	0.31
**LTC4S**	leukotriene C4 synthase	0.19	0.34	0.17	0.40
**MLYCD**	malonyl-CoA decarboxylase	0.15	0.37	0.16	0.44
**ALDH9A1**	aldehyde dehydrogenase 9 family member A1	0.15	0.38	0.13	0.45
**MAOA**	monoamine oxidase A	0.14	0.43	0.54	0.19
**ACAA2**	acetyl-CoA acyltransferase 2	0.13	0.44	0.11	0.45
**IL4I1**	interleukin 4 induced 1	0.10	0.50	0.20	0.38
**PSME1**	proteasome activator subunit 1	0.35	0.16	/	/
**ACSL1**	acyl-CoA synthetase long-chain family member 1	0.32	0.20	/	/
**GPD1**	glycerol-3-phosphate dehydrogenase 1	0.30	0.22	/	/
**AOC3**	amine oxidase, copper containing 3	0.24	0.24	/	/
**INMT**	indolethylamine N-methyltransferase	0.22	0.27	/	/
**MGLL**	monoglyceride lipase	0.22	0.28	/	/
**G0S2**	G0/G1 switch 2	0.21	0.30	/	/

**LGALS1**	galectin 1	0.20	0.32	/	/
**FMO1**	flavin containing monooxygenase 1	0.20	0.33	/	/
**DECR1**	2,4-dienoyl-CoA reductase 1	0.17	0.34	/	/
**UBE2L6**	ubiquitin conjugating enzyme E2 L6	0.17	0.35	/	/
**TP53INP2**	tumor protein p53 inducible nuclear protein 2	0.16	0.36	/	/
**CPT2**	carnitine palmitoyltransferase 2	0.15	0.36	/	/
**HSDL2**	hydroxysteroid dehydrogenase like 2	0.14	0.39	/	/
**GRHPR**	glyoxylate and hydroxypyruvate reductase	0.14	0.40	/	/
**BCKDHB**	branched chain keto acid dehydrogenase E1 subunit beta	0.14	0.40	/	/
**SUCLG1**	succinate-CoA ligase alpha subunit	0.14	0.41	/	/
**S100A10**	S100 calcium binding protein A10	0.14	0.42	/	/
**ACADS**	acyl-CoA dehydrogenase, C-2 to C-3 short chain	0.13	0.43	/	/
**HADHB**	hydroxyacyl-CoA dehydrogenase/3-ketoacyl-CoA thiolase/enoyl-CoA hydratase (trifunctional protein), beta subunit	0.13	0.44	/	/
**ECI1**	enoyl-CoA delta isomerase 1	0.12	0.45	/	/
**BPHL**	biphenyl hydrolase like	0.12	0.45	/	/
**PDHA1**	pyruvate dehydrogenase (lipoamide) alpha 1	0.12	0.46	/	/
**ACO2**	aconitase 2	0.12	0.47	/	/
**ACOT8**	acyl-CoA thioesterase 8	0.12	0.47	/	/
**HSD17B4**	hydroxysteroid 17-beta dehydrogenase 4	0.12	0.48	/	/
**ACAA1**	acetyl-CoA acyltransferase 1	0.11	0.48	/	/
**IDH3B**	isocitrate dehydrogenase 3 (NAD(+)) beta	0.11	0.49	/	/
**HMGCL**	3-hydroxymethyl-3-methylglutaryl-CoA lyase	0.11	0.48	/	/
**ADSL**	adenylosuccinate lyase	0.10	0.49	/	/
**HADH**	hydroxyacyl-CoA dehydrogenase	0.10	0.49	/	/
**IDH1**	isocitrate dehydrogenase (NADP(+)) 1, cytosolic	0.10	0.49	/	/
**ALAD**	aminolevulinate dehydratase	0.10	0.50	/	/

**IDH3G**	isocitrate dehydrogenase 3 (NAD(+)) gamma	0.09	0.50	/	/
**PTPRG**	protein tyrosine phosphatase, receptor type G	0.09	0.50	/	/
**SERINC1**	serine incorporator 1	0.09	0.51	/	/
**SUCLA2**	succinate-CoA ligase ADP-forming beta subunit	0.09	0.51	/	/
**HCCS**	holocytochrome c synthase	0.09	0.51	/	/
**NSDHL**	NAD(P) dependent steroid dehydrogenase-like	0.08	0.51	/	/
**NBN**	nibrin	0.08	0.52	/	/
**ADIPOR2**	adiponectin receptor 2	0.08	0.52	/	/
**ALDOA**	aldolase, fructose-bisphosphate A	0.08	0.52	/	/
**UROS**	uroporphyrinogen III synthase	0.08	0.52	/	/
**PCBD1**	pterin-4 alpha-carbinolamine dehydratase 1	0.08	0.53	/	/
**ACSS1**	acyl-CoA synthetase short-chain family member 1	0.08	0.53	/	/
**FASN**	fatty acid synthase	0.08	0.53	/	/
**ERP29**	endoplasmic reticulum protein 29	0.07	0.54	/	/
**LDHA**	lactate dehydrogenase A	0.07	0.54	/	/
**HMGCS2**	3-hydroxy-3-methylglutaryl-CoA synthase 2	/	/	0.75	0.07
**ACSM3**	acyl-CoA synthetase medium-chain family member 3	/	/	0.21	0.34
**EHHADH**	enoyl-CoA hydratase and 3-hydroxyacyl CoA dehydrogenase	/	/	0.17	0.42
**ACSL4**	acyl-CoA synthetase long-chain family member 4	/	/	0.13	0.42
**ACADM**	acyl-CoA dehydrogenase, C-4 to C-12 straight chain	/	/	0.13	0.44
**HSD17B7**	hydroxysteroid 17-beta dehydrogenase 7	/	/	0.11	0.44
**Neutral lipid metabolism**					
**Gene**	**Description**	**Rank metric score**	**Running ES**	**Rank metric score**	**Running ES**
**DGAT2**	diacylglycerol O-acyltransferase 2	0.45	0.07	0.40	0.30
**CAV1**	caveolin 1	0.37	0.13	0.20	0.48
**LIPE**	lipase E, hormone sensitive type	0.30	0.23	0.17	0.57
**CAV3**	caveolin 3	0.28	0.37	0.40	0.22

**MOGAT1**	monoacylglycerol O-acyltransferase 1	0.24	0.40	0.30	0.36
**SNCA**	synuclein alpha	0.21	0.46	0.65	0.14
**ABHD12**	abhydrolase domain containing 12	0.21	0.50	0.13	0.56
**SLC22A4**	solute carrier family 22 member 4	0.17	0.53	0.21	0.44
**ACSL1**	acyl-CoA synthetase long-chain family member 1	0.32	0.18	/	/
**GPD1**	glycerol-3-phosphate dehydrogenase 1	0.30	0.28	/	/
**ANG**	angiogenin	0.29	0.33	/	/
**MGLL**	monoglyceride lipase	0.22	0.43	/	/
**DGAT1**	diacylglycerol O-acyltransferase 1	0.18	0.51	/	/
**FABP4**	fatty acid binding protein 4	0.16	0.55	/	/
**AGPAT3**	1-acylglycerol-3-phosphate O-acyltransferase 3	0.15	0.57	/	/
**ABHD2**	abhydrolase domain containing 2	0.14	0.58	/	/
**PNPLA2**	patatin like phospholipase domain containing 2	0.13	0.58	/	/
**PNPLA3**	patatin like phospholipase domain containing 3	0.12	0.60	/	/
**LPIN1**	lipin 1	0.12	0.62	/	/
**GPD1L**	glycerol-3-phosphate dehydrogenase 1-like	0.11	0.62	/	/
**PLCE1**	phospholipase C epsilon 1	0.11	0.64	/	/
**AGMO**	alkylglycerol monooxygenase	/	/	0.25	0.40
**PCK1**	phosphoenolpyruvate carboxykinase 1	/	/	0.17	0.50
**FABP9**	fatty acid binding protein 9	/	/	0.17	0.53
**PCSK9**	proprotein convertase subtilisin/kexin type 9	/	/	0.13	0.59
**ACSL4**	acyl-CoA synthetase long-chain family member 4	/	/	0.13	0.61
**Lipid storage**					
**Gene**	**Description**	**Rank metric score**	**Running ES**	**Rank metric score**	**Running ES**
**DGAT2**	diacylglycerol O-acyltransferase 2	0.45	0.12	0.40	0.30
**PLIN5**	perilipin 5	0.41	0.23	0.22	0.59
**IL1B**	interleukin 1 beta	0.37	0.34	0.34	0.54
**CAV1**	caveolin 1	0.37	0.44	0.20	0.65
**ACVR1C**	activin A receptor type 1C	0.35	0.54	0.38	0.43

**CIDEA**	cell death-inducing DFFA-like effector a	0.33	0.63	0.52	0.17
**NRIP1**	nuclear receptor interacting protein 1	0.31	0.71	0.15	0.68
**ABHD4**	abhydrolase domain containing 4	0.25	0.77	/	/
**HEXB**	hexosaminidase subunit beta	0.23	0.83	/	/
**DGAT1**	diacylglycerol O-acyltransferase 1	0.18	0.85	/	/
**STAT5B**	signal transducer and activator of transcription 5B	/	/	0.13	0.70

Supplementary Table 3

Gene	Gene Name
CSA	also known as ERCC8, ERCC excision repair 8, CSA ubiquitin ligase complex subunit
CSB	also known as ERCC6, ERCC excision repair 6, chromatin remodeling factor
CUL4A	cullin 4A
DDB1	damage specific DNA binding protein 1
DDB2	damage specific DNA binding protein 2
ERCC1	ERCC excision repair 1, endonuclease non-catalytic subunit
GTF2H1	general transcription factor IIH subunit 1
GTF2H2	general transcription factor IIH subunit 2
GTF2H3	general transcription factor IIH subunit 3
GTF2H4	general transcription factor IIH subunit 4
GTF2H5	general transcription factor IIH subunit 5
LIG1	DNA ligase 1
MMS19	MMS19 homolog, cytosolic iron-sulfur assembly component
PCNA	proliferating cell nuclear antigen
POLD1	DNA polymerase delta 1, catalytic subunit
POLE	DNA polymerase epsilon, catalytic subunit
RAD23B	RAD23 homolog B, nucleotide excision repair protein
RFC1	replication factor C subunit 1
RPA1	eplication protein A1
XPA	XPA, DNA damage recognition and repair factor
XPB	also known as ERCC3, ERCC excision repair 3, TFIIH core complex helicase subunit
XPC	XPC complex subunit, DNA damage recognition and repair factor
XPD	also known as ERCC2, ERCC excision repair 2, TFIIH core complex helicase subunit
XPF	also known as ERCC4, ERCC excision repair 4, endonuclease catalytic subunit
XPG	also known as ERCC5, ERCC excision repair 5, endonuclease
UVSSA	UV stimulated scaffold protein A

Supplementary Table 4

**chrom**	chr4
**start**	1343466
**end**	1343473
**gene**	*UVSSA*
**ref**	AGTTCCT
**alt**	**A**
**type**	indel
**subtype**	del
**impact**	inframe_deletion
**codon change**	c.256_261del
**aa change**	p.Phe86_Leu87del
**impact severity**	MED
**median depth**	30
**mutated fraction**	100
